# Piezo1 induced apoptosis of type II pneumocytes during ARDS

**DOI:** 10.1186/s12931-019-1083-1

**Published:** 2019-06-11

**Authors:** Guo-Peng Liang, Jing Xu, Li-li Cao, Yi-Hua Zeng, Bai-Xu Chen, Jing Yang, Zhong-Wei Zhang, Yan Kang

**Affiliations:** 1 Department of Critical Care Medicine, West China School of Medicine and West China Hospital, Sichuan University, Chengdu, 610041 China; 2 Chengdu Medical College, Chengdu, 610041 Sichuan China; 3 Department of Respiratory Critical Care Medicine, West China School of Medicine and West China Hospital, Sichuan University, Chengdu, 610041 China

**Keywords:** Acute respiratory distress syndrome, Lung injury;Piezo1;apoptosis;Ca2+

## Abstract

**Objective:**

The mechanisms of lung injury in acute respiratory distress syndrome (ARDS) are not well understood.Piezo1 was recently identified as a mechanotransduction protein. The present study found the expression of Piezo1 in type II pneumocytes and investigated its role in mediating ARDS-related lung injury.

**Methods:**

Sprague-Dawley rats were used to establish an ARDS model, the expression of Piezo1,lung injuries, apoptosis as well as calcium influx were assessed.

**Results:**

Piezo1 was expressed in type II pneumocytes as shown by immunofluorescence staining and expression was increased in the ARDS model. Knockdown of Piezo1 reduced apoptosis which was related to the elevation of Bcl-2.Calcium influx played a vital role in Piezo1-induced apoptosis.

**Conclusion:**

Piezo1 was expressed in type II pneumocytes. Mechanical stretch of alveoli during ARDS induced activation of the Piezo1 channel,which resulted in calcium influx. The increased intracellular Ca2+ induced the apoptosis of type II pneumocytes, which may be related to the Bcl-2 pathway.

**Electronic supplementary material:**

The online version of this article (10.1186/s12931-019-1083-1) contains supplementary material, which is available to authorized users.

## Background

Acute respiratory distress syndrome (ARDS) is a common and life-threatening respiratory disorder of inflammatory lung injury responsible for 10% of all ICU admissions and a has a mortality rate of nearly 40% [1]. It is a type of acute diffuse, inflammatory lung injury, leading to increased pulmonary vascular permeability, increased lung weight, and loss of aerated lung tissue [2]. Recent evidence suggested that the main pathogenesis of ARDS included cytokine imbalance oxidative stress, and apoptosis of alveolar epithelial cells [3].However, the mechanisms of ARDS-related lung injury have not been clarified well.

Piezo1 was first detected in murine neuroblastoma cells and was recently identified as a component of mechanically-activated cation channels [4]. which were inherently mechanosensitive without relying on alternate cellular components for sensing mechanical stimuli [5]. Application of the force on cells caused calcium to rapidly enter cells through Piezo1 channels [6–8], which played an important role in sensing mechanical stretch. Piezo1 expressed in platelets, this contributed to Ca2+ entry and thrombus formation under arterial shear [9]. Piezo1 was found to be expressed in endothelial cells of developing blood vessels, and knockout of *Piezo1* was embryonically lethal with defects in vascular remodeling [10]. In addition,Piezo1 expressed in arterial smooth muscle cells was able to sense the fluid shear stress exerted by blood flow and affect arterial structure(diameter and wall thickness) by releasing ATP and regulating of NO formation to control vascular tone and blood pressure [11–13] . Piezo1 also senses extension of the bladder urothelium [14], intraluminal pressure changes and urine flow sensing [15]. These studies showed that Piezo1 is capable of detecting various kinds of mechanical stress and is involved in biological processes and maintenance of normal structures of human organs.

During respiration, alveoli and airways are stretched repetitively, and are constantly under mechanical stress generated by air and the surrounding tissues. Respiratory disorders tend to enhance mechanical stretch by a series of pathophysiological mechanisms, that lead to lung injury. Mechanical ventilation is another important factor that may increase lung injury due to tidal hyperinflation [16, 17], and cyclic recruitment/derecruitment [18]. Given that Piezo1 is a mechanosensitive ion channel with the ability to sense mechanical stretch, we assumed that Piezo1 was also expressed in the lung and may be a potential mechanism of lung injury. The mRNA expression profile indicated that Piezo1 may be expressed in the lung [4]. However, to the best of our knowledge, no studies have identified the physiological functions of Piezo1 in lung injury.

Therefore, the present study was designed to investigate the expression and functions of Piezo1 in the lung and study the potential mechanisms of Piezo1 in ARDS-related lung injury. We attempted to find a new target for prevention and treatment of ARDS.

## Materials and methods

### Animals and experimental design

The protocol was approved by the Institutional Animal Care and Treatment Committee of Sichuan University (Chengdu, P.R. China). Health adult Sprague-Dawley (SD) rats weighing 250 ± 10 g(Experimental Animal Center of Academy of Military Medical Sciences of the People’s Liberation Army, China) were housed in cages with free access to water and food and divided randomly into four groups: 1) control group, 2)ARDS group, 3)ARDS+ high tidal volume(HV)group and 4)ARDS+ low tidal volume(LV) group,(five rats per group). Control rats were injected with 0.1 mL/kg body weight physiological saline while those in experimental groups were given 0.1 mL/kg body weight oleic acid by intravenous injection and the rats were spun around 10 times to establish ARDS model which was then confirmed by assessing partial pressure of oxygen in the arterial blood after 3 h. ARDS+HV and ARDS+LV rats were ventilated with a tidal volume of 10 mL/kg and 6 mL/kg respectively,with positive end-expiratory pressure (PEEP) 3 cmH2O, and respiratory frequency of 25/min for 4 h. Next, rats in all groups were anaesthetized, and lung sections were collected.

### Assessment of lung injury

Blood(50ul) was collected from aorta abdominalis and partial pressure of oxygen and carbon dioxide(PaO2,and PaCO2,respectively)was measured by blood gas analyzer (Abbott i-STAT). Oxygenation index was calculated as PaO2/FiO2, and lung morphology was observed by hematoxylin and eosin(HE)staining of left lung tissue. The wet/dry ratio (W/D ratio) was calculated to reflect the severity of pulmonary edema. Wet weights of right lower lungs were measured upon collection of lung tissue, and then lungs were incubated in an oven (60 °C for 72 h) to measure dry weight. The levels of serum TNF-α and IL-1β were measured by ELISA assay(MD6692,MD6693).

### Cell culture and transfection

Human lung epithelial cell line A549 was seeded on 10 cm dishes in DMEM, and cultured at 37^°C^ and 5% CO2. Cells were transiently transfected with either Piezo1 siRNA or control siRNA using Lipofectamine 2000(Invitrogen, Carlsbad, CA, USA;11,668,019),the sequence of siRNA is provided in Additional file 1: Figure S2-A.

### RNA isolation, real-time q-PCR, and western blots

After 24-48 h post transfection, total RNA was extracted using Trizol(Invitrigen)following the manufacturers’ instructions. Complementary DNAs (cDNAs) were synthesized using the HiScript® II One Step RT-PCR Kit (Vazyme Biotech, Nanjing, China)and amplified using specific primers for *Piezo1*.qPCR was performed with a LightCycler 480 system(Roche, Basel, Switzerland). GAPDH was used as a reference gene for relative quantification of *Piezo1* gene expression. The primer sequences are as follows:

*Piezo1*-F 5′-GCTGTACCTGCCTGATTTCTTCC-3′.

*Piezo1*-R 5′-CATGCGCTGCCACTCCTCT-3′;

*GAPDH*-F 5′-CACATGGCCTCCAAGGAGTAA-3′; and.

*GAPDH*-R 5′-TGAGGGTCTCTCTCTTCCTCTTGT-3′.

Western blots were performed as previously described [19]. Antibodies included Piezo1 (Santa Cruz Biotechnology, Dallas, TX, USA; sc-164,319),dilution ratio 1:1000),Bcl-2(Abgent, San Diego, CA, USA, dilution ratio 1:1000),caspase3 + cleaved caspase3(Cell Signaling Technology, Danvers, MA, USA; 9664 t, dilution ratio 1:2000) and GAPDH (Abcam, Cambridge, UK; Ab8245; dilution ratio 1:1000).

### Isolation of lung type II pneumocytes

Type II pneumocytes were isolated from SD rats by a modified method as previously described [20]. Briefly, the cannula was inserted into the pulmonary artery through the right ventricular and the right lung was lavaged with normal saline supplemented with heparin for 2–3 min, then the heart and lungs were quickly severed together with the trachea and taken out from the body. Lavage was performed through trachea cannula using normal saline(5 mL× 8), then 2 ml PBS containing 0.1% collagenase and 0.125% trypsin was infused into the right lung and the lung was incubated the lung in 5% CO_2_ at 37^°C^ for 25 min. The lung was cut into small pieces and FBS was used to inactivate trypsin, then the lung was transferred into 20 mL PBS containing 1 mL DNAse I, and incubated in a 37^°C^ water bath for 5 min. Lung single suspension were filtered through 60,150 and 280 mesh filters in turn and the supernatant was discarded after centrifugation. The pellet was resuspended in DMEM media and seeded in 10 cm dish for 1 h. Then, the unadhered cells were collected and transferred into another dish. Finally, the cells were identified by immunofluorescence staining of pulmonary surfactant protein C(SP-C),the marker of type II pneumocytes.

### Immunofluorescence and immunohistochemistry

Expression of Piezo1 was first examined by immunohistochemical staining in lung tissues and then examined by immunofluorescence staining. For immunohistochemistry, paraffin-embedded and dewaxed lung sections were exposed to citrate buffer for antigen unmasking, then blocked for 30 min using BSA. Sections were incubated overnight at 4 °C with Piezo1 and SP-C primary antibodies, then incubated with horseradish peroxidase (HRP) for 30 min the next day. For immunofluorescence, lung sections were prepared in the same manner but incubated with secondary antibodies at 37^°C^ for 30 min the next day. Then, sections were stained with DAPI (Beyotime,Shanghai,China;C1002) and the images were captured using a fluorescence microscope(Leica, Wetzlar, Germany;DM500) .

### Apoptosis assay

For primary cells derived from SD rats, single cell suspensions were prepared as mentioned in the “Isolation of lung type II pneumocytes cells” section. For cell lines, A549 cells were digested by trypsin without EDTA. The cell concentration was adjusted to 3 × 10 [5]/mL and stained with a 100ul binding solution containing 5ul annexin V-fluorescein isothiocyanate and 5ul PI solution in a 100ul binding solution. Apoptosis was assayed using a Novo Cyte flow cytometry (Acea Biosciences, San Diego, CA, USA) .

### Mechanical stretch and calcium imaging

BioFlex culture plates were coated with type I collagen in 4 °C for 16-24 h. A549 (5 × 10 [5])cells were then seeded on the plates for 24 h, and fresh serum-free medium was used before applying mechanical stretch. FX-4000 (Flexercell Corporation, Burlington, NC, USA) was used to produce mechanical stretch on cells with a square wave.The mechanical force intensity was set up as 15%,frequency 0.5HZ,and lasted for 4 h. The control group was subjected to the same procedure but without stretch stimulation. After the experiment, cells were collected and washed twice with PBS and resuspended in 1 mL PBS, and then 500ul of 10 μM calcium probes (Fluo-3/AM, Beyotime; S1056) was added into cell suspensions and incubated in the dark at 37^°C^ for 30 min. Confocal imaging (TCS SP8,Leica,German) was performed to detect Ca2 + .Fluo-3 AM fluorescence signals were measured at 488 nm excitation wavelength and 528 nm emission wavelength. We chose eight visual fields and calculated the fluorescence intensity and cell counts with Image Pro Plus 6.0.The calcium signal was calculated as total fluorescence intensity/ cell counts.

### Statistical analysis

The data were analyzed using SPSS 19.0 software and expressed as mean ± standard error. The results in different groups were compared by one-way ANOVA and post hoc Tukey was used for multiple comparisons. *P* < 0.05 was considered statistically significant.

## Results

### The expression of Piezo1 in type II pneumocytes

**Fig. 1:**
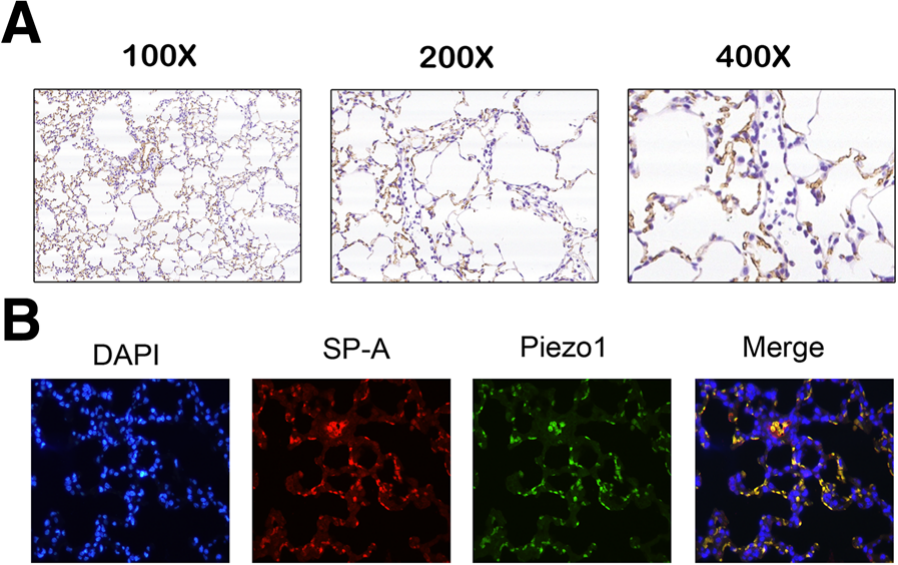
The expression of Piezo1 in type II pneumocytes. **a** Immunohistochemical staining of Piezo1 in rat lung tissue. Piezo1 positive cells stained in brown. **b** Expression of Piezo1 in Type II pneumocytes(scale bar:20um).Type II pneumocytes were labelled in red with a specific marker(SP-C).Piezo1 positive cells stained in green. SP-C and Piezo1 double staining depicted in orange, indicated Piezo1 was expressed in type II pneumocytes

From immunohistochemical staining, Piezo1 was found to be expressed in the rat lung, mainly located around the alveoli(Fig. 1a). As type II pneumocytes are one of the components of alveolar epithelia and have a crucial role in maintaining regular alveolar morphology, we speculated whether Piezo1 was expressed in type II pneumocytes. Surfactant protein C (SP-C) was used to specifically label type II pneumocytes by immunofluorescence staining. Double-staining showed that Piezo1-positive cells were mostly positive for SP-C(Fig. 1b), confirming that Piezo1 was predominantly expressed in type II pneumocytes.

### Piezo1 induced apoptosis of type II pneumocytes in ARDS

**Fig. 2:**
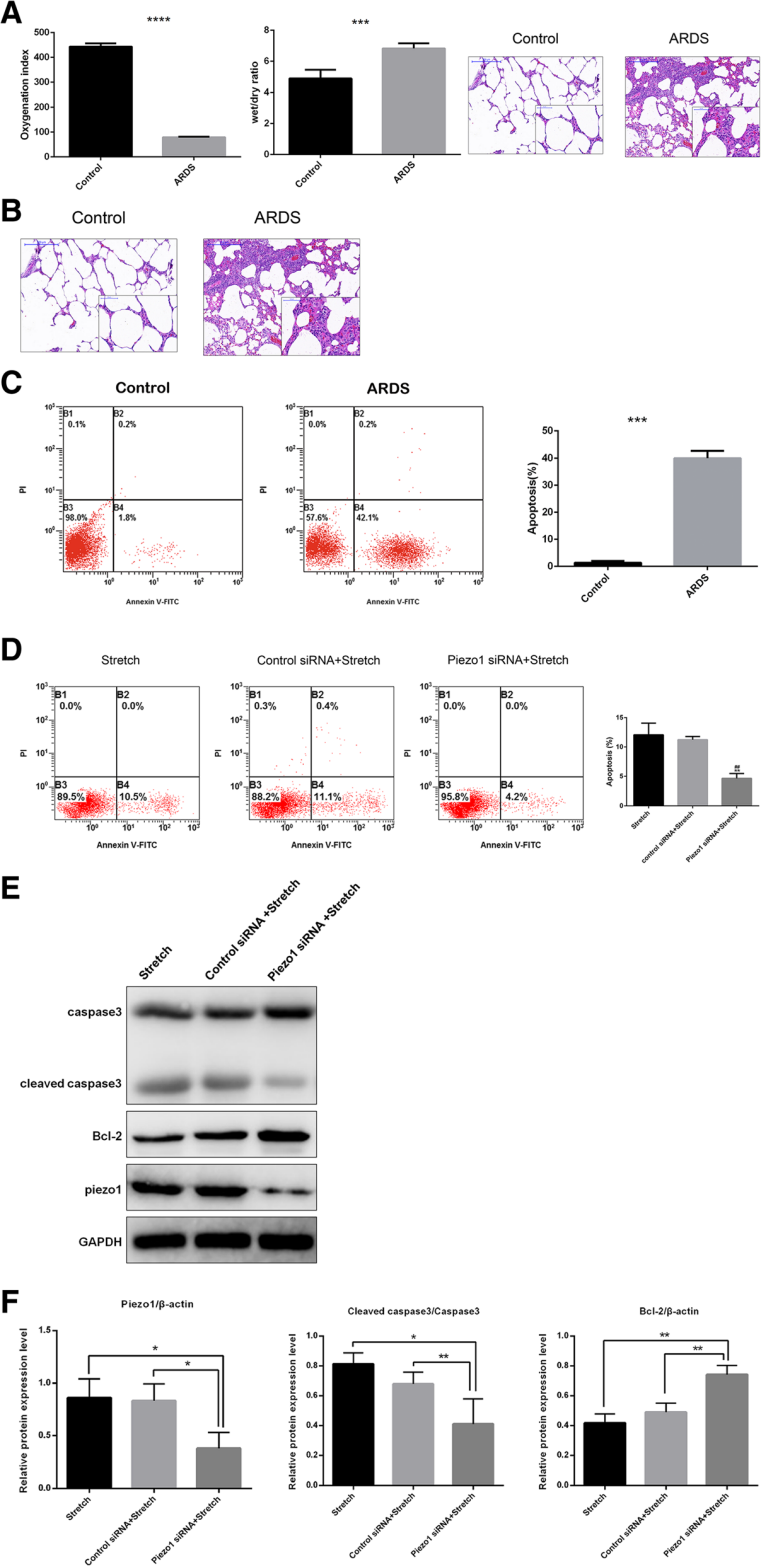
Piezo1 induced type II pneumocytes apoptosis in ARDS. **a** Oxygenation index was calculated as PaO2/FiO2, OI was less than 300 in ARDS as defined by Berlin definition. Wet/dry ratio was significantly increased in ARDS rat. Lung morphometry assessed by HE staining in different groups (100X/400X magnification) edema, hemorrhage and alveolar wall thickness were observed in the image of ARDS group accompanied by inflammatory cells infiltration .**b** Immunofluorescence staining of Piezo1and SP-A. Piezo1 and SP-A double positive cells stained in orange.(100X magnification) **c** Apoptosis of type II pneumocytes in control and ARDS mice, analyzed by annexin V-fluorescein isothiocyanate/PI double staining. Cells in the B2 and B4 quadrants(annexin V+/PI+ and annexin V+/PI-, respectively) were considered to be apoptotic. Graphical representation shows significant difference. **d**. Apoptosis of A549 cells treated with stretch and/or siRNA, analyzed by annexin V-fluorescein isothiocyanate/PI double staining. Graphical representation shows significant difference. **e** Western blot of Piezo1, caspase 3 and Bcl-2 in different group. Stretch represents A549 under stretch, control siRNA + stretch represents A549 transfected with control siRNA and was applied stretch.Piezo1 siRNA + stretch represents knock down of Piezo1 in A549 and applied stretch. Cleaved caspase 3 was the activated form of caspase. GAPDH was used as internal reference. **f** The relative protein expression levels of cleaved caspase3/caspase3,Bcl-2 and Piezo1

To investigate the role of Piezo1 in ARDS, the oleic acid-induced ARDS model was established in rats and confirmed by assessing oxygenation index, wet/dry ratio, HE staining(Fig. 2a) and other indicators(Additional file 1: Figure S1A) We next compared Piezo1 expression between control and ARDS rats, and found that Piezo1 was significantly increased in the ARDS group(Fig. 2b), indicating that Piezo1 may play a role in ARDS progression. As Piezo1 was mainly expressed in type II pneumocytes, and numerous studies have provided evidence that apoptosis of type II pneumocytes is one of the mechanisms of ARDS, we sought to determine whether Piezo1 mediated type II pneumocytes apoptosis in ARDS. First, we isolated type II pneumocytes and confirmed by immunofluorescence staining of SP-C(Additional file 1: Figure S1B).Then we analyzed the apoptosis of type II pneumocytes from control and ARDS rats by Annexin V/PI staining with flow cytometry. Consistent with other studies, the apoptosis of type II pneumocytes was markedly increased in ARDS rats in our study(Fig. 2c).

To test whether this apoptosis was partly due to the upregulation of Piezo1, alveolar epithelial cells A549 were transfected with silent RNA to knock down *Piezo1* expression. The efficacy of knockdown was assessed by qPCR(Additional file 1: Figure S2A). Because Piezo1 is a “stretch sensing” protein, as reported before, and one of the mechanisms of ARDS is stress-induced lung injury, we applied stretch to A549 cells to mimic the mechanical stretch between individual alveoli due to heterogeneous compliance in ARDS. The apoptosis induced by mechanical stretch was intensity-dependent and 15% mechanical stretch could induce moderate apoptosis(Additional file 1: Figure S2B),thus, we used this intensity in the subsequent experiments. It was shown that knockdown of *Piezo1* reduced stretch-induced apoptosis (Fig. 2d).

To further confirm that Piezo1 mediates apoptosis and determine the pathway involved, we performed western blot to assay the apoptosis-related proteins. Caspase-3 is activated in apoptotic cells both by extrinsic (death ligand) and intrinsic (mitochondrial) pathways and Bcl-2 is localized to the outer membrane of mitochondria and regulates intrinsic apoptosis. From western blot analysis, Piezo1 was successfully knocked down in A549, accompanied by decreased cleavage of caspase-3 (Fig. 2e, f) and increased Bcl-2 (Fig. 2e, f). These results demonstrated that Piezo1 mediated type II apoptosis of pneumocytes in ARDS through the Bcl-2 pathway.

### Piezo1 mediated apoptosis depends on calcium influx

**Fig. 3:**
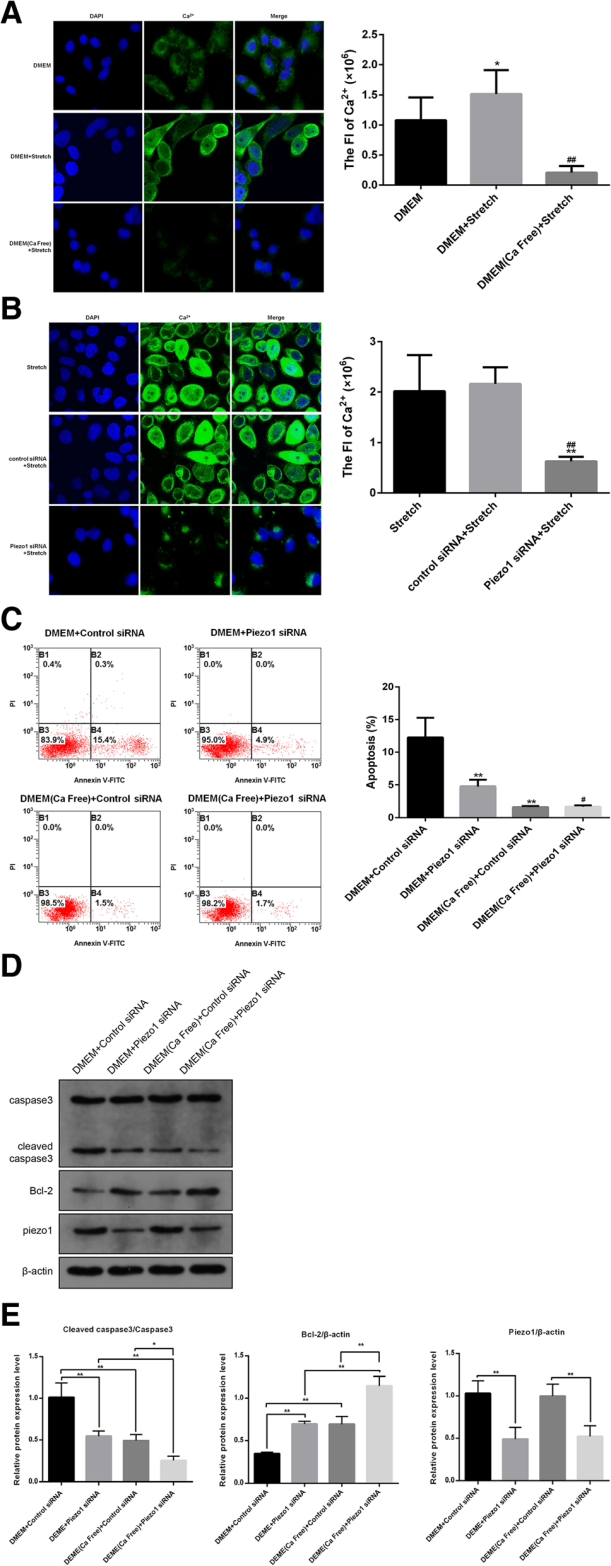
Piezo1 mediated apoptosis depends on calcium influx. **a** A549 cells were treated with 15% stretch with a frequency of 0.5HZ for 4 h. Ca2+ was labeled with10 μM Fluo-3/AM and observed by confocal imaging. Selected eight visual fields randomly and detected total fluorescent intensity by Image pro Plus 6.0.Calculatedsingle cell fluorescent intensity as total fluorescent intensity/cell counts. In Ca2+ free group, calcium was removed from medium. (1000 × magnification) **b** A549 was transfected with Piezo1-siRNA72hrsbefore treated with mechanical stretch and Ca2+ influx was assayed by confocal imaging. (1000 × magnification) **c** The apoptosis of A549 treated with mechanical stretch was assessed by flow cytometry and annexin V-fluorescein isothiocyanate/PI double staining. **p < 0.01.vs. DMEM +Control siRNA; ##*p* < 0.01.vs. DMEM+Piezo1 siRNA. **d** The expression of apoptosis related proteins are assayed by western blot. **e** The relative protein expression levels of cleaved caspase3/caspase3,Bcl-2 and Piezo1

Our observations indicated that Piezo1-mediated apoptosis was related to Bcl-2. Bcl-2 proteins were critical regulators of intracellular Ca2+ dynamics [21] whereby Piezo1 was a selected cation channel that controls stretch induced Ca2+ influx [22]. Therefore, we speculated that activated Piezo1 during stretch increased Ca2+ influx and induced apoptosis. To verify the this, we assayed Ca2+ influx by detecting the fluorescence intensity of Ca2 + .Stretch increased intracellular Ca2+ concentration and this elevation was absent when Ca2+ was deprived in medium(Fig. 3a), demonstrating that the elevated Ca2+ was due to Ca2+ influx rather than Ca2+ release from storage. Notably, we observed that knockdown of Piezo1 dramatically reduced Ca2+ influx during stretch(Fig. 3b) The apoptosis was of no significant change between the cells with or without Piezo1 knockdown when Ca2+ was deprived(Fig. 3d, e) We next assessed the expression of caspase 3 and Bcl-2 of A549 in Ca2+ free medium. Expression of Bcl-2 increased in Ca2+ free condition, indicating the negative relationship between Ca2+ and Bcl-2 (Fig. 3d, e).

### Piezo1 is a potential mechanism to explain the protective role in lung injury of low tidal volume ventilation

**Fig. 4:**
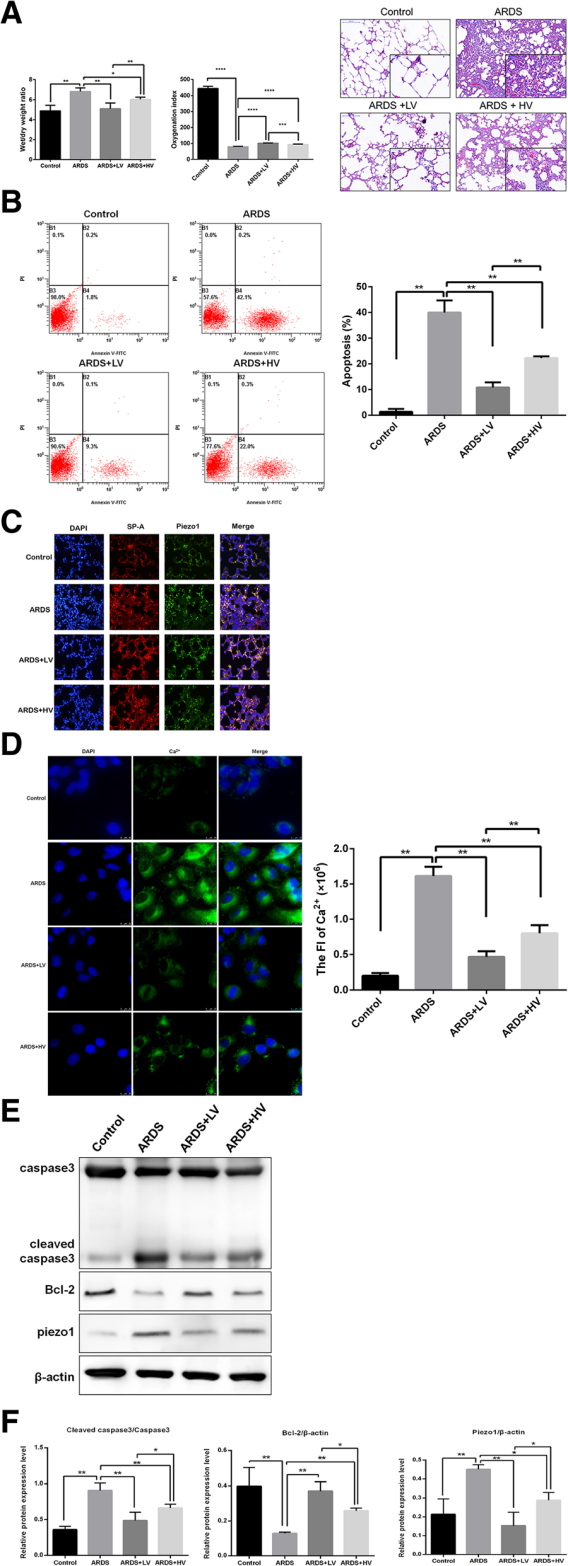
Piezo1 was a potential mechanism to explain the protective role in lung injury of LTV. **a** Wet/dry ratio decreased and oxygenation index (calculated as PaO2/FiO2) in different group. Lung morphometry assessed by HE staining (100X/400X magnification) **b** The apoptosis of type II pneumocytes assayed by flow cytometry. **c** Immunofluorescence staining of Piezo1 and SP-A. Piezo1 and SP-A double positive cells stained in orange.(100 × magnification) **d** Ca2+ influx detected by confocal microscope and the quantitation of immunofluorescence presented as a bar graph. (1000 × magnification) **e** The expression of caspase 3,Bcl-2 and Piezo1 in rats assessed by western blot (cleaved caspase 3 was an activated form of caspase 3) (NS, not significant. *, *p* < 0.05.**, *p* < 0.001.LV low tidal volume, HV high tidal volume.) **f** The relative protein expression levels of cleaved caspase3/caspase3,Bcl-2 and Piezo1

Low tidal volume ventilation(LTV) has been recommended as a lung protective strategy in ARDS. However, the mechanisms are not well clarified. LTV uses a low volume ventilation (4-6cmH2O) with a proper PEEP to avoid lung overdistension and opens collapsed alveoli. This reduces ventilation homogeneity, and thus alleviates stretch forces in lung parenchyma at margins between aerated and atelectatic regions. Therefore, we examined whether Piezo1 explained part of this function of LTV by ventilating oleic acid injected rats with either LTV(Vt 6cmH2O/kg, PEEP 3cmH2O)or high tidal volume ventilation HVT(Vt 15cmH2O/kg, PEEP 0cmH2O).Rats with LTV presented less severe ARDS and lung injury(Fig. 4a Additional file 1: Figure S3) In addition, the apoptosis of type II pneumocytes was significantly reduced in LTV rats compared with HVT rats(Fig. 4b) Notably, we observed less expression of Piezo1 in LTV rats. In line with the relationship between Piezo1 and Ca2+ influx we’ve revealed above, rats ventilated with low volume had reduced Ca2+ influx (Fig. 4d) and caspase 3 accompanied with elevated Bcl-2 (Fig. 4e)These results support that LTV-protected lungs may partly be due to the regulation of Piezo1.

## Discussion

The mechanisms of ARDS have not been well clarified to-date, and a lack of specific pharmacotherapies has lead to high mortality. Here for the very first time, we have found Piezo1, expressed in type II pneumocytes of rats which may be likely potential targets for treatment of ARDS. Furthermore, we demonstrated that Piezo1-mediated apoptosis of type II pneumocytes via increased calcium influx during ARDS. In addition, our data revealed a potential mechanism of low tidal volume ventilation in alleviating lung injury through regulating Piezo1 expression.

Alveolar epithelium injury contributes significantly to the development of acute pulmonary edema which eventually results in ARDS-related lung injury [23]. As one of the components of alveolar epithelia, type II pneumocytes have many functions, including surfactant production [24], immune function [25] as well as clearance of alveolar edema fluid [26] We observed that Piezo1 in type II pneumocytes was upregulated during ARDS, and our results as well as that of other published studies [27–30] indicated that apoptosis of type II pneumocytes increased and played a vital role in lung injury. Therefore, we assumed that Piezo1 mediated the apoptosis of type II pneumocytes. To verified this, we used mechanical stretch to establish an in vitro model since the inhomogeneous stress of alveoli in ARDS contributed a big part to the process, besides, as a mechanical ion channel sensing various types of mechanical stretch [6, 11, 31–35], Piezo1 was induced by the mechanical stretch. Consistent with our hypothesis, we found that knockdown of *Piezo1* dramatically reduced the apoptosis of type II pneumocytes and we also found it was related to Bcl-2, the proteins that mediate the process by which mitochondria contribute to cell death known as the intrinsic apoptosis pathway [21] In line with our results, it was reported that stretch-induced apoptosis of type II pneumocytes was mediated by the intrinsic mitochondrial pathway rather than extrinsic pathway [36].

Furthermore, we found Piezo1 induced apoptosis in a calcium-dependent way.Piezo1 was new family of mechanosensitive ion channels that allow transmembrane Ca2+ flux into cells, they were associated with the physiological response to touch, pressure, and stretch [14]. Naohiko Murata et al. found mechanical stretch induced Ca2+ influx in human lung fibroblast, which was not caused by activation of conventional stretch-sensitive ion channels [37]. We demonstrated that *Piezo1* knockdown reduced Ca2+ influx during stretch rather than Ca2+ release from internal Ca2+ storage. Furthermore, removal of Ca2+ diminished Piezo1 mediated apoptosis. These suggested that Ca2+ influx was likely to be a “second message” followed by activation of Piezo1 that signaled the apoptosis pathway. Ca2+ overload played a vital role in inducing apoptosis [38]. In our results, we found that Piezo1-induced apoptosis was related to Bcl-2, and the diminish of Ca2+ increased Bcl-2 expression to a level comparable to Piezo1 knockdown. These results implies the apoptosis generated by Piezo1-induced Ca2+ influx may be associated with Bcl-2 change. It was unveiled that Bcl-2 regulated calcium homeostasis and protected cells from apoptosis by reducing Ca2+ overload [21]. Therefore, Bcl-2 may involve in Piezo1-induced apoptosis by functioning as a calcium regulated protein. Christopher J. Winters et.al revealed that CAMKII inhibition blunted bleomycin-induced apoptosis in type II pneumocytes by decreasing cytoplasmic Ca2+ concentration, the potential mechanism related to endoplasmic reticulum (ER) Ca2+ release. Bcl-2 was demonstrated to reduce Ca2+ by competing for the ligand-binding of Ca2+ channel in ER [39]. Thus it was possible that CAMKII promotes the signaling pathway of Ca2 + −induced apoptosis whereas Bcl-2 inhibits this process.

Patients with ARDS tend to have rapid and shallow breathing patterns due to a decreased lung volume, this promotes more intensified stress due to the increase of cyclic alveolar collapse and reopening, leading to upregulation of Piezo1.Mechanical ventilation is a common supportive therapy of ARDS, which relieves dyspnea and improves oxygen in patients. However, mechanical ventilation has also been shown to significantly contribute to aggravating lung injury, called ventilation induced lung injury(VILI),especially when used with inappropriately high tidal volumes [26]. Therefore, low tidal volume ventilation was recommended as a lung protective strategy to avoid alveolar overdistention. We compared LTV and HVT in our rat model and found that Piezo1 expression along with apoptosis and Ca2+ influx was significantly reduced when low tidal volume was used. This supports that Piezo1 may be a potential mechanism to explain the protective role of LTV.

## Conclusions

The results of our studies demonstrated that Piezo1 played an pivotal role in lung injury. Activation of Piezo1 by mechanical stretch induced the apoptosis of type II pneumocytes via Ca2+ influx. Inhibition of Piezo1 or Ca2+ influx may become a potential target for preventive or therapeutic interventions aiming at relieving lung injury in ARDS. The Piezo1 mediated apoptosis was related to Bcl-2, but further research is required to support this.

## Additional file

Additional file 1: **Figure S1.** The expression of Piezo1 on rat type II pneumocytes. A)The level of IL-1β and TNF-a increased in ARDS rats, tested by ELISA assay. B)Identification of Type II pneumocytes isolated from lung. Nuclei stained with DAPI, Positive staining depicted in blue. SP-C containing cells stained in green. SP-C is a specific marker for type II pneumocytes(1000 × magnification). **Figure S2.** Piezo1 induced type II pneumocytes apoptosis in ARDS.A)The efficacy of knockdown of piezo1 was evaluated by Q-PCR and siRNA-1 was used in the following experiments. B) The apoptosis of A549 cells strength of mechanical stretch, assayed by annexin V-fluorescein isothiocyanate/PI double staining. **Figure S3.** Piezo1 was a potential mechanism to explain the protective role in lung injury of LTV. The level of IL-1βand TNF-a in different groups, tested by ELISA assay. (DOCX 605 kb)

## Data Availability

The datasets used and/or analyzed during the current study are available from the corresponding author on reasonable request.

## Abbreviations

ARDS: acute respiratory distress syndrome
HE: hematoxylin and eosin
IL-1β: interleukins-1β
MV: mechanical ventilation
PEEP: positive end-expiratory pressure
SD: Sprague-Dawley
SP-A: pulmonary surfactant protein A
TNF-α: tumor necrosis factor-a
W/D: wet-to-dry ratio
