# Supplementary material for: Piezo1 induced apoptosis of type II pneumocytes during ARDS
Source: Respir Res. 2019 Jun 11;20:118. doi: 10.1186/s12931-019-1083-1 (PMC6558715; doi:10.1186/s12931-019-1083-1)
Supplement: Supplementary file 1 — Figure S1. The expression of Piezo1 on rat type II pneumocytes. A)The level of IL-1β and TNF-a increased in ARDS rats, tested by ELISA assay. B)Identification of Type II pneumocytes isolated from lung. Nuclei stained with DAPI, Positive staining depicted in blue. SP-C containing cells stained in green. SP-C is a specific marker for type II pneumocytes(1000 × magnification). Figure S2. Piezo1 induced type II pneumocytes apoptosis in ARDS.A)The efficacy of knockdown of piezo1 was evaluated by Q-PCR and siRNA-1 was used in the following experiments. B) The apoptosis of A549 cells strength of mechanical stretch, assayed by annexin V-fluorescein isothiocyanate/PI double staining. Figure S3. Piezo1 was a potential mechanism to explain the protective role in lung injury of LTV. The level of IL-1βand TNF-a in different groups, tested by ELISA assay. (DOCX 605 kb) [file 12931_2019_1083_MOESM1_ESM.docx]

**Additional file 1**


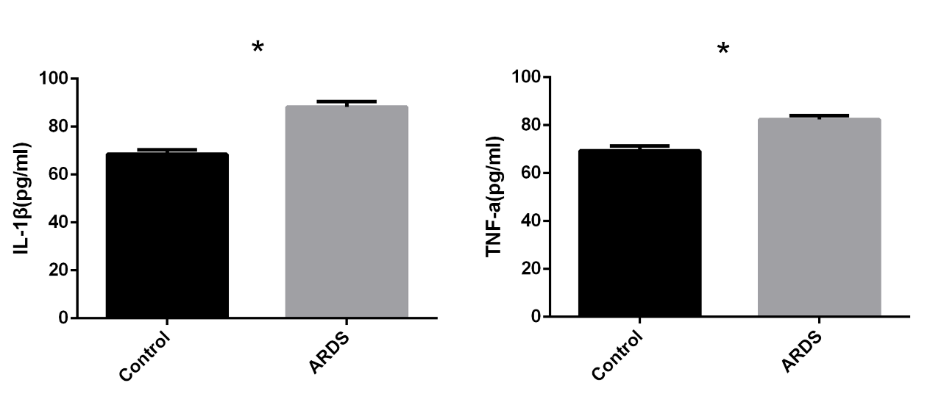


A


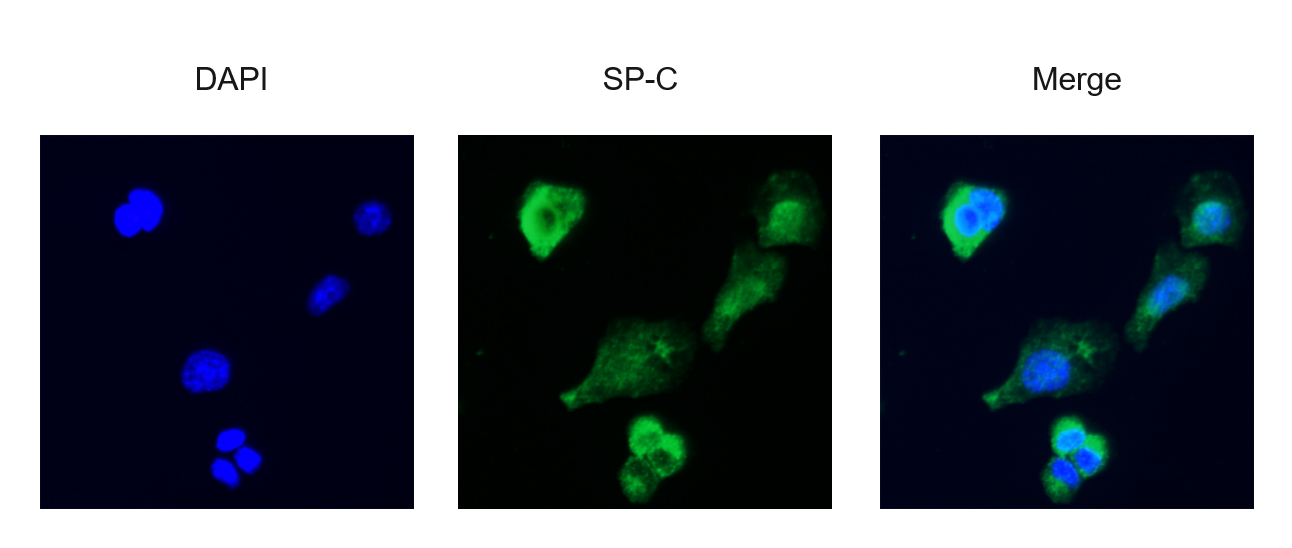


B

**Figure S1.** The expression of Piezo1 on rat type II pneumocytes. A)The level of IL-1β and TNF-a increased in ARDS rats, tested by ELISA assay. B)Identification of Type II pneumocytes isolated from lung. Nuclei stained with DAPI, Positive staining depicted in blue. SP-C containing cells stained in green. SP-C is a specific marker for type II pneumocytes(1000×magnification).

**
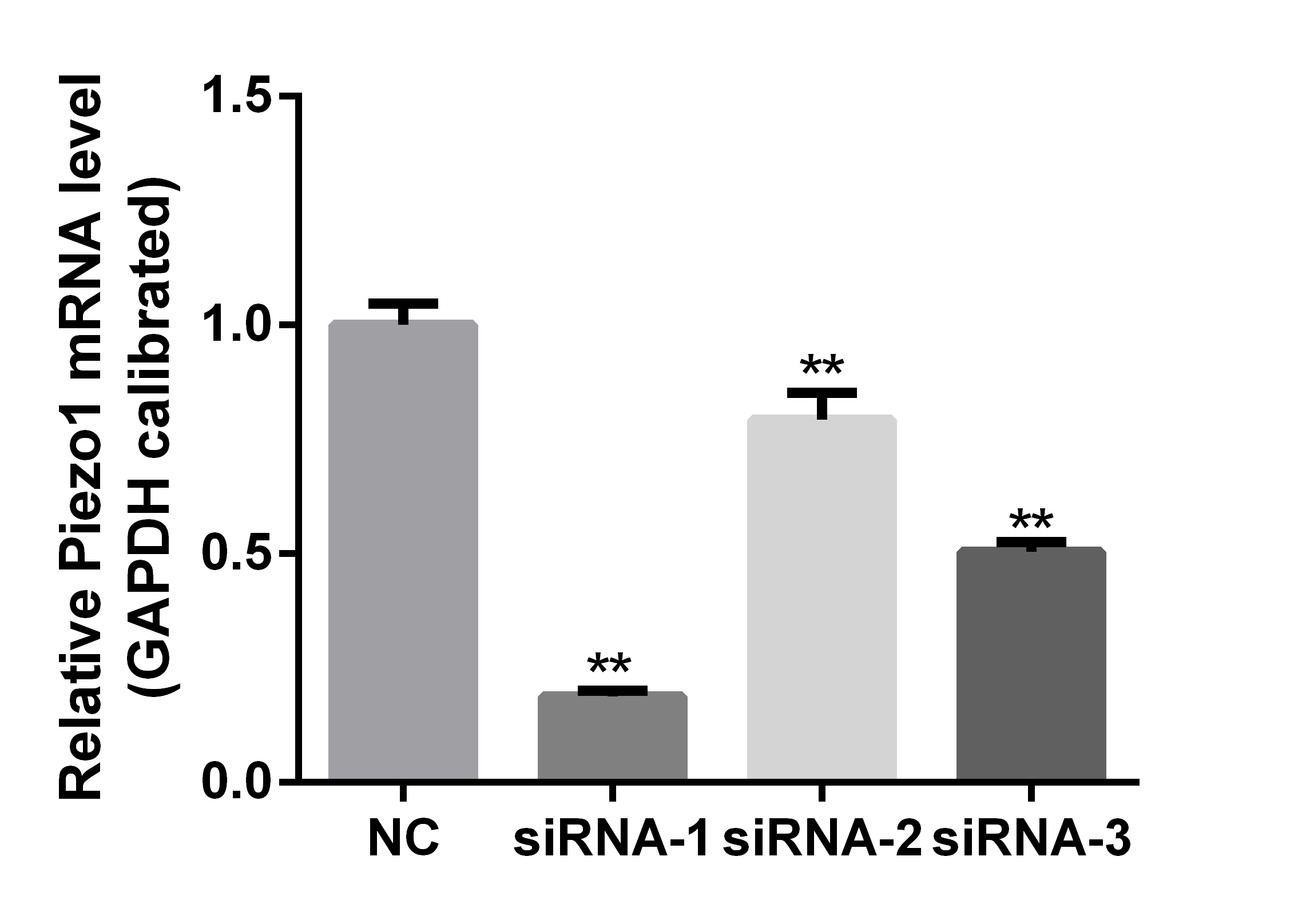
**

A


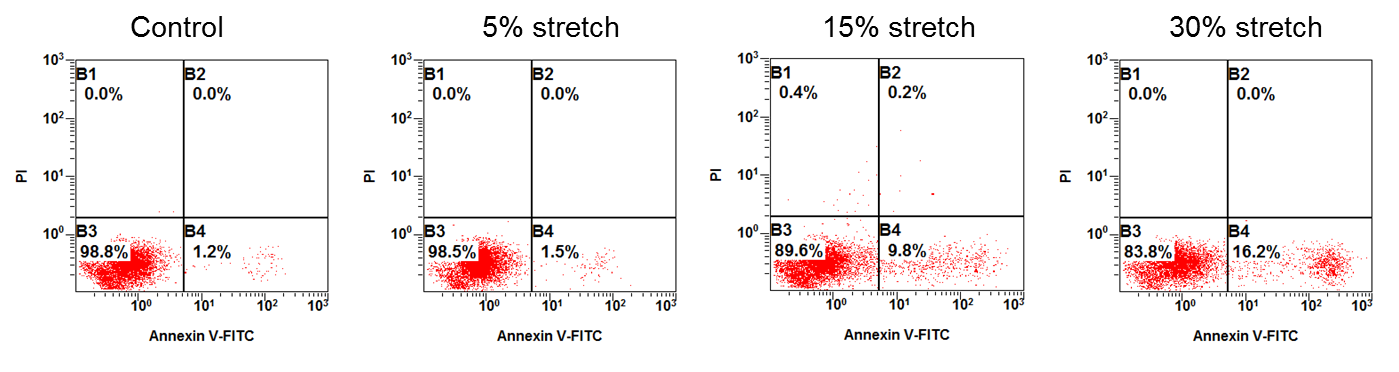


B

**Figure S2.** Piezo1 induced type II pneumocytes apoptosis in ARDS.A)The efficacy of knockdown of piezo1 was evaluated by Q-PCR and siRNA-1 was used in the following experiments. B) The apoptosis of A549 cells strength of mechanical stretch, assayed by annexin V-fluorescein isothiocyanate/PI double staining.


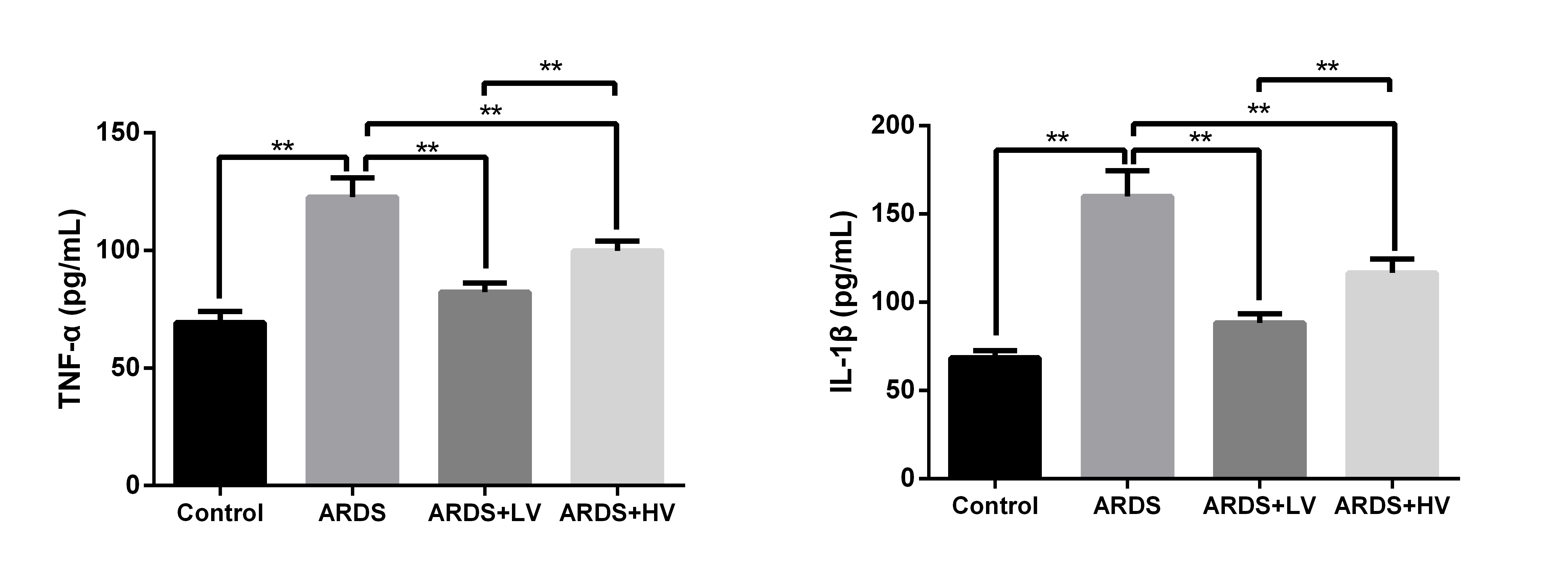


**Figure S3.** Piezo1 was a potential mechanism to explain the protective role in lung injury of LTV. The level of IL-1βand TNF-a in different groups, tested by ELISA assay.
